# Using 6S-5-methyltetrahydrofolate instead of folic acid in prenatal multivitamin reduces unmetabolized folic acid concentrations in the mother-fetus dyad: a 24-week randomized controlled trial

**DOI:** 10.3389/fnut.2026.1679067

**Published:** 2026-03-26

**Authors:** Fulvia Draicchio, Jeanette Hausser, Mastaneh Sharafi, Adeline Grier-Welch, Arianne Vance, Nima Alamdari, Itamar D. Futterman, Scott Chudnoff, Olga Malysheva, Marie A. Caudill, Isabelle Hanson, Xinyin Jiang

**Affiliations:** 1 Department of Health and Nutrition Sciences, Brooklyn College of the City University of New York, Brooklyn, NY, United States; 2 Ritual, Los Angeles, CA, United States; 3 Department of Obstetrics and Gynecology, Division of Maternal Fetal Medicine, Maimonides Medical Center, Brooklyn, NY, United States; 4 Division of Nutritional Sciences, Cornell University, Ithaca, NY, United States

**Keywords:** 5-methyltetrahydrofolate, folic acid, pregnancy, prenatal multivitamin, unmetabolized folic acid

## Abstract

A growing number of prenatal multivitamin (MVI) products use 5-methyltetrahydrofolate (5-MTHF) in place of the synthetic folic acid (FA), because FA needs to be reduced to 5-MTHF for use in cellular methylation reactions and excess FA consumption can increase unmetabolized FA (UMFA) concentrations in circulation. However, the efficacy of 5-MTHF supplementation on maintaining maternal and fetal folate status in comparison to FA has not been sufficiently investigated. This study examined whether using 6S-5-MTHF instead of FA in a prenatal MVI affected total folate and UMFA status in maternal and fetal tissues. Eighty first-trimester pregnant participants were randomized to receive either a prenatal MVI containing 1,000 μg DFE of 6S-5-MTHF (MTHF-MVI group) or 1,330 μg DFE of FA (FA-MVI group) for 24 weeks (*n* = 40/group) beginning at their second trimester. Fasting blood samples were collected at week 0, 12, and 24 of the study whereas cord blood and placental samples were collected at delivery. Thirty-one participants in each group completed the study. By week 24 of the study, 5-MTHF and total folate concentrations in maternal and cord serum and the placenta did not differ between groups. There were significantly fewer participants (7% vs. 31%) with detectable UMFA and lower average UMFA concentrations in maternal blood of the MTHF-MVI group vs. the FA-MVI group (*p* < 0.01). UMFA concentrations in the placenta were lower in the MTHF-MVI group than the FA-MVI group (*p* < 0.01). Few cord blood samples (*n* = 2) contained detectable UMFA and both were from the FA-MVI group. In conclusion, during the second and third trimesters of pregnancy, prenatal MVIs providing 6S-5-MTHF may be an efficacious alternative to FA to maintain total folate status while reducing UMFA concentrations in maternal and fetal compartments. More research is needed to delineate the influence of different folate forms on fetal health and developmental outcomes.

**Clinical trial registration:**

ClinicalTrials.gov, identifier: NCT05673070.

## 1 Introduction

Folate is a water-soluble B vitamin naturally found in a wide range of foods, particularly in dark green leafy vegetables, fruits and their juices, beans, nuts, grains, eggs, seafood, and grains. A series of randomized controlled trials (RCTs) have consistently demonstrated that folic acid (FA) supplementation during the periconceptional period can reduce the risk of neural tube defects (NTDs) of fetuses (1). As a result, mandatory FA fortification in enriched cereal grain products was implemented in the United States in 1998 and continues to the present day (2). FA is the synthetic, oxidized form of folate, which is metabolically inactive. For FA to be used in biological processes, it must be reduced by dihydrofolate reductase (DHFR) sequentially to form tetrahydrofolate (THF). THF can then be converted to different forms of folate such as 5, 10-methylene-THF, 10-formyl-THF, and 5-methyltetrahydrofolate (5-MTHF), and participate in nucleotide synthesis and the methionine cycle for methyl group transfer reactions (3).

FA supplementation as low as 200 μg in combination with normal intake of foods fortified with FA has been found to cause increases in unmetabolized folic acid (UMFA) in the blood. Further, some animal and *in vitro* studies indicate that high UMFA status may inhibit folate metabolizing enzymes such as DHFR and methyltetrahydrofolate reductase (MTHFR) (4, 5). However, the clinical implications of UMFA elevation in humans remain unclear (6–8).

It is recommended that women capable of becoming pregnant or who are pregnant consume 400 μg of FA per day to reduce the risk of NTDs (1). However, the benefits of FA supplementation throughout the remainder of pregnancy beyond neural tube closure (which typically occurs around day 28 of gestation), remain less well understood (9). Specifically, questions remain regarding the necessity of continued high FA intake later in pregnancy, particularly in FA-fortified populations where cumulative intake may be excessive.

Studies that examine how supplementation of other forms of folate, such as 5-MTHF, during mid and late pregnancy influences maternal and fetal folate status and health outcomes are limited. Nevertheless, there are an increasing number of prenatal multivitamin mineral (MVI) supplements on the market that use the metabolically active 5-MTHF form of folate instead of FA, representing the growing interest in these products among both women trying to conceive and those who are pregnant (10). The gap in knowledge regarding the safety and comparative effectiveness of taking supplements containing different forms of folate for the mother-fetus dyad is apparent. Pharmacokinetic studies demonstrate that the bioavailability of 5-MTHF may be slightly higher than FA initially, yet this difference may disappear after a few days of supplementation. Additionally, the response may vary among different populations (11–13). A 2024 RCT demonstrated that pregnant women receiving 625 μg of either 5-MTHF (*n* = 30) or FA (*n* = 30) as a supplement daily for 16 weeks showed no difference in their red blood cell or serum folate status (14). However, how placental and fetal folate status may be affected by different forms of folate in prenatal MVI supplements remains largely unknown.

Given the unresolved questions regarding the health impact of folate forms during preconception and pregnancy, RCTs are necessary to clarify how these differing folate forms influence the folate status of the mother-fetus dyad, especially considering possible interaction with other nutrients in a prenatal MVI. Therefore, the current study aimed to compare the differential effects of using a prenatal MVI containing 5-MTHF vs. FA on maternal and fetal folate status, UMFA concentrations, and DNA methylation beyond the first trimester of pregnancy.

## 2 Materials and methods

### 2.1 Study population and study procedures

The study was a 24-week double-blind RCT. Pregnant individuals (*n* = 80) who received prenatal care at the Maimonides Medical Center in Brooklyn, NY, USA were recruited to participate in the study from October 2023 to October 2024. Inclusion criteria included English-speaking, over 18 years of age, gestational age between 12 and 16 weeks, and singleton pregnancy. Exclusion criteria were chronic illnesses including diabetes, cardiovascular conditions including hypercholesterolemia and hypertension that required medical treatment, kidney disease, and liver disease. Using simple randomization, participants were assigned to either take a prenatal multivitamin (Essential for Women Prenatal Multivitamin, Ritual, USA) providing 1,000 μg dietary folate equivalents (DFE) of 6S-5-MTHF (approximately 588 μg of 6S-5-MTHF based on a conversion factor of 1.7) per serving (*n* = 40) (MTHF-MVI group) or a standard control prenatal multivitamin, which provided 800 μg of FA (approximately 1,330 μg DFE) (*n* = 40) (FA-MVI group). While the two MVIs did not contain equivalent amounts of folate, they were matched as closely as possible using commercially available options in order to mimic the real-world circumstances facing pregnant individuals. It should be noted that the two MVIs had other differences in composition, such as the content of other B vitamins, choline, and vitamin D, which are detailed in Supplementary Table 1. Participants took their assigned MVI daily for 24 weeks during the second and third trimesters of pregnancy.

At enrollment, each participant completed a baseline questionnaire which included demographic and medical information. Three 24-h dietary recalls, two on weekdays and one on a weekend day, were obtained from each participant by a trained research assistant via phone following the baseline visit (15). In addition to dietary recalls, blood samples were collected at baseline, and at study weeks 12 and 24, corresponding to gestational weeks 24 and 36, respectively. Prior to study visits, participants were instructed to fast for at least 4 h, then 10 mL of blood was drawn into a serum separator SST tube (BD, Franklin Lakes, NJ, USA) to retrieve serum and an EDTA BD vacutainer tube to retrieve plasma and buffy coat. Adherence to the protocol was assessed by a pill count at the second and third visits.

Immediately after delivery, cord venous blood was collected to retrieve plasma, buffy coat, and serum. Full thickness placenta biopsies (0.5 × 0.5 cm) were retrieved at the center of four virtually divided quadrants of the placental disk and pooled for analysis (16). These pooled placental samples were either fixed with RNAlater^®^ (Thermo Fisher, Suwanee, GA, USA) or homogenized in liquid nitrogen. All samples were stored at −80°C until analysis. Obstetric and birth outcome information including neonate sex, mode of delivery, and birth weight was collected through medical chart review.

The study protocol was reviewed and approved by both the City University of New York (CUNY) Institutional Review Board (IRB) (Approval ID:2022-0670) and the Maimonides Medical Center IRB (Approval ID: 2023-08-07). Written informed consent was obtained from each participant before participation in the study. The trial was registered on ClinicalTrials.gov (NCT05673070).

### 2.2 Sample size calculation

The sample size calculation was based on detecting a 40% difference in UMFA concentrations between groups at 80% power and a 0.05 significance level. A 25% attrition rate was also factored in the calculation given the high mobility of the urban population, to reach the *n* = 40/group sample size. The sample size chosen was considered as generally sufficient for similar pilot RCTs (17).

### 2.3 Diet analysis

Dietary data was analyzed with the Nutrition Data System for Research (NDSR) software to analyze nutrient intake. Average daily intakes were calculated as the average daily consumption of nutrients over the three days of dietary recalls (18). Dietary recalls with extremely low (< 500 kcal/day) or high (>5,000 kcal/day) total energy intake were excluded from data analysis. Total folate intake was calculated as the sum of folate intake from both diet and supplements. The tolerable upper intake level (UL) for folate is 1,000 μg FA which does not include intake from naturally-occurring food folates. To compare whether a participant's intake exceeded the UL, the European Food Safety Authority (EFSA) interpretation of UL was used (19), that is, in addition to FA consumed from fortified foods and the FA-MVI supplement, folate as 6S-5-MTHF from the MTHF-MVI supplement was also added after being converted to μg FA equivalent. Since an official conversion factor has not been established, a conversion factor of 1.7 that assumes similar bioavailability of 5-MTHF and FA was used. The sum of FA and FA equivalent from fortified food and supplement was compared to the UL (19).

### 2.4 Analytical measurements

#### 2.4.1 Folate quantification

Serum and placenta folate species were measured by liquid chromatography mass spectrometry liquid chromatography-mass spectrometry (LC-MS)/MS using published methods (20). Folates were quantified using Thermo QExactive mass spectrometer operating in PRM positive mode with Vanquish UPLC.

#### 2.4.2 Genotyping

DNA was extracted from the cord blood samples using the GeneJET Genomic DNA Purification Kit (Thermo Fisher) following manufacturer's instructions. Amplification for the target SNP MTHFR C677T (rs1801133) was performed using ThermoFisher Scientific TaqMan assays with pre-designed TaqMan probes (Thermo Fisher). Real-time PCR condition was 95°C for 10 min, followed by 40 cycles of 95°C for 15 s and 60 C for 1 min and fluorescence of the two probes FAM and VIC, labeling the two alleles of a SNP, was detected and analyzed by the genotyping program in the BioRad CFX Manager software.

#### 2.4.3 Global DNA methylation

Global methylation was assessed after nuclease P1, alkaline phosphatase, and phosphodiesterase (Sigma-Aldrich, St. Louis, MO) trienzyme treatment of the genomic DNA samples, followed with measurements using the DNA methylation ELISA kit (Cayman Chemical, Ann Arbor, Michigan, USA) according to the manufacturer's instructions.

### 2.5 Statistical analyses

Demographic characteristics at baseline were compared using Student's *t*-test for continuous variables and χ-square test for nominal variables. To compare the differences in folate intakes and placental and cord blood measurements, a generalized linear model was used with intervention groups as the independent variable and controlling for a predefined set of covariates including maternal age, race/ethnicity, education levels, parity, sex of the neonate, gestational age, and maternal pre-pregnancy BMI initially (21). The covariates were dropped from the model in a step-wise process if they did not reach statistical significance. For maternal serum folates, a general linear mixed model was used with group and week of visit as independent variables and participant code as a nested variable. The maternal demographics and folate levels at baseline were also included as covariates. Maternal demographic variables were removed from the model in a step-wise process when they did not reach statistical significance. Data that were not normally distributed were log-transformed before analyses. Data were presented as mean ± standard deviation (SD). A *p* value of < 0.05 was considered significant. Data were analyzed with SPSS software (version 24, IBM Inc., Armonk, NY, USA).

## 3 Results

### 3.1 Demographic characteristics

**Figure 1:**
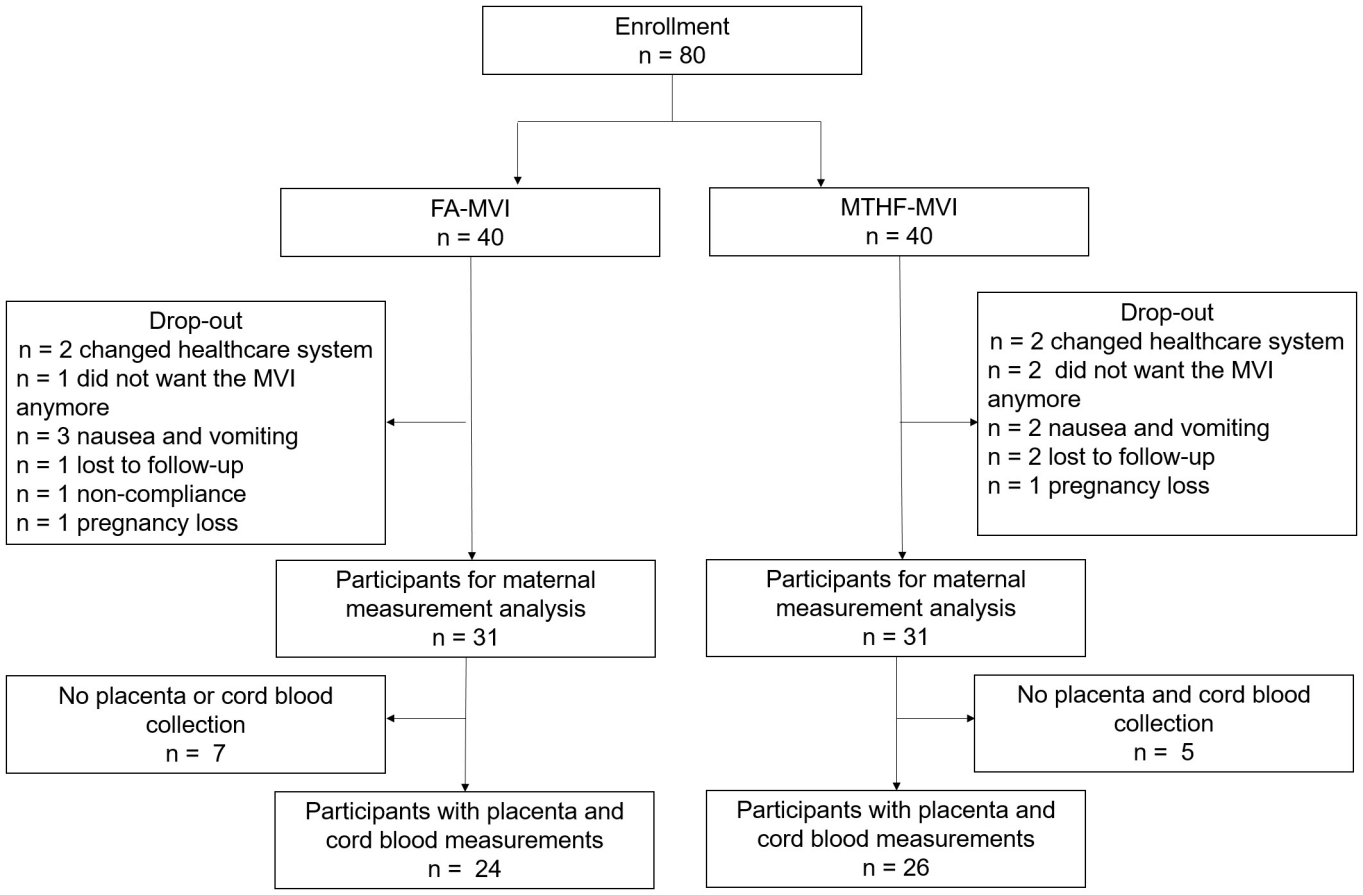
Study flow chart. FA, folic acid; MTHF, methyltetrahydrofolate; MVI, multivitamin.

Of the total of 80 participants enrolled in the study, 18 dropped out and 62 completed the study, including 31 from the MTHF-MVI group and 31 from the FA-MVI group. Reasons for drop-out included nausea and vomiting and other symptoms related to pregnancy (*n* = 5), transferring to another healthcare group (*n* = 4), pregnancy loss or congenital defects unrelated to study activities (*n* = 2), lost to follow-up (*n* = 3), non-compliance (*n* = 1), and not feeling comfortable to take supplements from a study (*n* = 3) (Figure 1). There were no significant differences in the number of drop-outs between groups for any of the reasons. Since no data or samples were collected beyond the baseline for participants who dropped out, we only included the 62 participants who completed the study for per-protocol final data analyses. At baseline, there were no differences in most demographic or anthropometric characteristics such as maternal age, prepregnancy BMI, race/ethnicity, education levels, prior tobacco and alcohol use, current vitamin use, and parity between the two groups. However, there were more participants that were married in the FA-MVI vs. the MTHF-MVI group (*p* = 0.03) (Table 1).

**Table 1:** Participant demographics.

**Participant information**	**MTHF-MVI (*n* = 31)**	**FA-MVI (*n* = 31)**	***p*-value**
Age (year)	31.2 ± 5.5	32.4 ± 5.5	0.40
Prepregnancy BMI (kg/m2)	27.7 ± 6.2	26.1 ± 5.0	0.27
Race			0.84
White	9 (29%)	12 (38.7%)	
Black	9 (29%)	9 (29%)	
Asian	8 (25.8%)	6 (19.4%)	
Other	5 (16.1%)	4 (12.9%)	
Ethnicity			0.79
Hispanic	10 (32.3%)	11 (35.5%)	
Non-Hispanic	21 (67.7%)	20 (64.5%)	
Education			0.63
Less than or equal to high school	5 (16.1%)	8 (25.8%)	
Some college	22 (71%)	20 (64.5%)	
College graduate and higher	4 (12.9%)	3 (9.7%)	
Marital status			0.03^*^
Single	17 (54.8%)	8 (25.8%)	
Married	13 (41.9%)	23 (74.2%)	
Separated	1 (3.2%)	0 (0%)	
Prior tobacco use	2 (6.5%)	2 (6.5%)	0.30
Prior alcohol use	8 (25.8%)	6 (19.4%)	0.77
Current prenatal vitamin user	27 (87.1%)	24 (77.4%)	0.32
Nulliparity	11 (35.5%)	13 (41.9%)	0.60

Analyzed with χ-square test for categorical variables and Student's *t*-test for continuous variables (data were presented as mean and standard deviation). ^*^p < 0.05 is considered as significantly different from the FA-MVI group.

### 3.2 More FA-MVI participants had a supplemental folate intake exceeding the UL

All dietary intake data were collected after enrollment and randomization to one of the two MVIs. There were no differences in the dietary intake of natural folate and FA from fortified food between groups at week 0 and week 12. At week 24, the MTHF-MVI group had 30% lower natural food folate intakes (*p* = 0.033) (Table 2). After accounting for folate intake from prenatal MVIs, the MTHF-MVI group had lower total folate intakes than the FA-MVI group (*p* = 0.001). Nevertheless, all participants had intake levels above the folate RDA of 600 μg DFE for pregnant women. However, at the end of the 24-week study, 29% of participants in the FA-MVI group had FA intake levels exceeding the UL of 1,000 μg throughout the study period while none of the MTHF-MVI participants had supplemental and fortified folate intakes exceeding the UL.

**Table 2:** Folate intake of study participants.

**Folate intake**	**MTHF-MVI (*n* = 31)**	**FA-MVI (*n* = 31)**	***p*-value**	**Difference or RR**	**95% CI**
Dietary natural folate (μg DFE)				Difference	
Week 0	188 ± 117	144 ± 72	0.076	44	−5, 94
Week 12	151 ± 75	203 ± 205	0.19	−52	−130, 27
Week 24	143 ± 54	206 ± 148	0.033^*^	−63	−121, −5
**FA from fortified food (**μ**g)**					
Week 0	156 ± 154	189 ± 245	0.53	−33	−137, 71
Week 12	140 ± 137	194 ± 206	0.23	−54	−142, 35
Week 24	145 ± 139	196 ± 203	0.27	−51	−141, 50
**Total folate intake from diet and supplement (**μ**g DFE)**					
Week 0^a^	1454 ± 268	1825 ± 405	< 0.001^*^	−371	−546, −197
Week 12	1357 ± 327	1893 ± 500	< 0.001^*^	−536	−750, −321
Week 24	1345 ± 344	1777 ± 627	< 0.001^*^	−432	−688, −330
Participants with fortified and supplement folates exceeding UL (*n*,%)^b^						RR	
Week 0	0	5 (16%)	0.053	0.091	0.0052, 1.58
Week 12	0	8 (26%)	0.005^*^	0.059	0.0035, 0.98
Week 24	0	9 (29%)	0.001^*^	0.051	0.0031, 0.84

^a^Week 0 data were collected after enrollment and randomization to receive one of the two multivitamins. ^b^Based on the European Food Safety Authority (EFSA) interpretation of UL, 6S-5-MTHF from supplement (1,000 μg DFE) was converted to μg FA equivalent using a conversion factor of 1.7 assuming similar bioavailability of 5-MTHF and FA. The sum of FA and FA equivalent from fortified food and supplement was compared to the UL (19). Analyzed with χ-square test for categorical variables and Student's *t*-test for continuous variables (data were presented as mean and standard deviation). ^*^p < 0.05 is considered as significantly different from the FA-MVI group. CI, confidence interval; FA, folic acid; MTHF, methyltetrahydrofolate; MVI, multivitamin; RR, relative risk; UL, upper tolerable intake level.

### 3.3 More participants had UMFA in their blood in the FA-MVI group

Upon examination of the serum folate metabolites in the two groups, there were no differences in 5-MTHF or total folate (FA+5-MTHF) concentrations between groups in the maternal blood throughout the study (Table 3). There were no changes in the concentrations of these metabolites over time eithe*r*. At the end of the study, 12 and 17 participants had supraphysiologic concentrations (>20 ng/mL) of total folate (22), concentrations that are higher than the physiologic need, in the two groups, respectively. There were no differences in total folate status between groups (*p* = 0.21). Serum UMFA was detectable among 26% and 32% of participants in the MTHF-MVI and FA-MVI groups at week 0, respectively, which was not significantly different (*p* = 0.78). The MTHF-MVI group had a drop in FA detection rate over time which was not observed in the FA-MVI group. As a result, at week 24 there were significantly more participants with detectable UMFA (31% vs. 7% detection rate) and significantly higher average UMFA concentrations in the FA-MVI group vs. in the MTHF-MVI group (*p* = 0.013) (Table 3).

**Table 3:** Maternal serum folate metabolites.

**Serum folate**	**MTHF–MVI (*n* = 31)**	**FA–MVI (*n* = 31)**	**Difference or RR**	**95% CI**	***p* group**	***p* week**	***p* group × week interaction**
Serum total folate (ng/mL)			Difference		0.16	0.08	0.77
Week 0	21.5 ± 6.6	22.0 ± 8.8	−0.5	−4.5, 3.4			
Week 12	21.5 ± 7.0	24.4 ± 11.0	−2.9	−7.6, 1.8			
Week 24	19.7 ± 7.0	22.0 ± 6.5	−2.3	−5.8, 1.1			
Serum methyltetrahydrofolate (ng/mL)					0.99	0.14	0.52
Week 0	21.0 ± 6.2	20.8 ± 6.6	0.2	−3.1, 3.4			
Week 12	21.5 ± 7.0	22.7 ± 9.3	−1.2	−5.4, 3.0			
Week 24	19.9 ± 7.0	20.7 ± 6.6	−1.6	−4.9, 1.7			
Unmetabolized folic acid (ng/mL)					0.014^*^	0.39	0.27
Week 0	0.5 ± 1.4	1.2 ± 3.5	−0.7	−2.1, 0.7	0.32		
Week 12	0.06 ± 0.3	1.8 ± 4.6	−1.7	−3.3, −0.04	0.044^*^		
Week 24	0.02 ± 0.1	0.8 ± 1.7	−0.7	−1.3, −0.2	0.013^*^		
Presence of unmetabolized folic acid (*n*, %)			RR		0.001^*^	0.63	0.63
Week 0	8 (26%)	10 (32%)	0.80	0.36, 1.75	0.78		
Week 12	2 (6%)	11 (35%)	0.18	0.04, 0.75	0.011^*^		
Week 24	2 (7%)	9 (31%)	0.22	0.25, 0.91	0.041^*^		

Analyzed with mixed model controlling for baseline folate metabolite levels. ^*^p < 0.05 is considered as significantly different. The serum total folate concentration is calculated as folic acid + methyltetrahydrofolate concentrations. CI, confidence interval; FA, folic acid; MTHF, methyltetrahydrofolate; MVI, multivitamin; RR, relative risk.

### 3.4 The MTHFR C677T genotypes did not have a clear effect on maternal blood folate metabolites in either group

The potential modifying effect of the *MTHFR* C677T (rs1801133) genotype on maternal blood folate metabolites and their response to the two different MVIs were also assessed (Table 4). The *MTHFR* C677T SNP did not result in different 5-MTHF or total folate concentrations at any time point in either group. However, at baseline those with the TT genotype had significantly higher concentrations of UMFA than those with the CC and CT genotypes (*p* < 0.05), yet such differences were eliminated at week 12 and 24 in both groups.

**Table 4:** Folate levels in participants with different MTHFR C677T genotypes.

**Serum folate**	**CC (*n* = 23)**	**CT (*n* = 20)**	**TT (*n* = 19)**	**CT vs. CC**		**TT vs. CC**		***p* genotype**	***p* group × genotype interaction**
				**Difference**	**95% CI**	**Difference**	**95% CI**		
**Serum methyltetrahydrofolate (ng/mL)**									
Week 0	22.4 ± 7.4	19.3 ± 4.9	20.7 ± 6.1	−3.2	−7.0, 0.6	−1.7	−5.6,2.2	0.24	0.37
Week 12	23.1 ± 10.8	20.7 ± 6.0	22.1 ± 6.5	−2.4	−7.5, 2.6	−1.0	−6.1, 4.1	0.59	0.98
Week 24	19.8 ± 6.5	21.2 ± 6.2	20.5 ± 7.1	1.5	−2.5, 5.5	0.7	−3.4, 4.8	0.86	0.98
**Serum total folate (ng/mL)**									
Week 0	22.8 ± 7.6	19.5 ± 5.1	22.8 ± 9.8	−3.3	−8, 1.4	0.06	−4.7,4.8	0.26	0.49
Week 12	23.3 ± 10.9	22.0 ± 7.2	23.6 ± 9.6	−1.3	−7.0, 4.5	0.3	−5.6, 6.1	0.80	0.83
Week 24	20.0 ± 6.7	22.1 ± 6.8	20.6 ± 7.1	2.1	−2.1, 6.3	0.6	−3.6, 4.9	0.75	0.90
**Unmetabolized folic acid (ng/mL)**									
Week 0^a^	0.33 ± 0.52	0.25 ± 0.46	2.10 ± 4.72	−0.08	−1.53, 1.70	1.77	0.73, 3.40	0.047^*^	0.36
Week 12	0.13 ± 0.35	1.34 ± 2.99	1.39 ± 5.14	1.21	−0.82, 3.24	1.26	−0.80, 3.31	0.44	0.45
Week 24	0.22 ± 0.77	0.86 ± 1.89	0.12 ± 0.37	0.63	−0.09, 1.36	−0.10	−0.84, 0.64	0.25	0.26

Analyzed with generalized linear model with intervention group and the rs1801133 genotype as independent variables; ^a^This model controlled for prepregnancy BMI additionally. ^*^p < 0.05 is considered as significantly different. The serum total folate concentration is calculated as folic acid + methyltetrahydrofolate concentrations. CI, confidence interval.

### 3.5 Placental UMFA concentrations were higher in the FA-MVI group

Next, folate metabolites in the full thickness placental samples (which contained both the maternal and fetal tissues and blood) and cord blood were measured. There were three major forms of folate metabolites in the placenta, including 5-MTHF, FA, and 5-formyl-THF. 5-MTHF (*p* = 0.71) and total folate metabolite (*p* = 0.33) concentrations did not differ between groups. Both 5-formyl-THF (*p* = 0.012) and UMFA (*p* < 0.01) concentrations were higher in the FA-MVI group vs. the MTHF-MVI group (Table 5). Accordingly, the UMFA detection rate was significantly higher in the FA-MVI group than the MTHF-MVI group (*p* < 0.01).

**Table 5:** Placenta and cord blood folate metabolite and DNA methylation levels.

**Folate status**	**MTHF-MVI**	**FA-MVI**	**Difference**	**95% CI**	***p*-value**
**Placenta**					
5-MTHF (ng/g tissue)	412 ± 95	422 ± 81	−10	−60, 41	0.71
UMFA (ng/g tissue)	0.52 ± 0.63	2.93 ± 2.13	−2.41	−3.29, −1.53	< 0.01^*^
5-formyl THF (ng/g tissue)	29.4 ± 9.6	43.6 ± 25.1	−14.2	−24.8, −3.5	0.01^*^
Total folate (ng/g tissue)	442 ± 95	469 ± 91	−26	−79, 27	0.33
Global DNA methylation (fold difference)	0.7 ± 0.5	1.0 ± 1.0	−0.3	−0.8, 0.1	0.14
**Cord blood**					
Serum 5-MTHF (ng/mL)	32.1 ± 9.2	40.8 ± 17.1	−8.7	−14.6, 0.5	0.08
Global DNA methylation (fold difference)	0.7 ± 0.4	1.0 ± 0.5	−0.3	−0.5, 003	0.08

Analyzed with generalized linear models controlling for maternal folate intake at week 24. FA, folic acid; 5-MTHF, 5-methyltetrahydrofolate. ^*^p < 0.05 is considered as significantly different from the FA-MVI group. The placental total folate concentration is calculated as FA + 5-MTHF + 5-formyl THF concentrations. CI, confidence interval; FA, folic acid; MTHF, methyltetrahydrofolate; MVI, multivitamin; UMFA, unmetabolized folic acid.

The main form of folate detected in the cord blood was 5-MTHF. There were no differences in cord blood 5-MTHF levels between groups (*p* = 0.08) (Table 5). Two cord blood samples had detectable UMFA, both of which belonged to the FA-MVI group.

### 3.6 Global DNA methylation levels

Global DNA methylation was measured in the placenta and cord blood, but no significant differences were found between the two groups (*p* = 0.14 and 0.082, respectively) (Table 5).

## 4 Discussion

The National Institutes of Health (NIH) identified knowledge gaps related to the differential effects of 5-MTHF vs. FA supplementation during pregnancy on folate status and functional outcomes of the mother-fetus dyad in a 2019 expert workshop (23), yet studies that address these questions are still limited. In this RCT, supplementation with a prenatal MVI containing 6S-5-MTHF, as compared to FA, resulted in reduced concentrations of UMFA in both the maternal and placental compartments while having no significant difference in total folate status. These results suggest that 5-MTHF is an efficacious form of folate for pregnant women and their fetuses in the second and third trimesters of pregnancy.

FA is the predominant and recommended form of folate to be used for women are pregnant or may become pregnant, mainly for its important role in reducing NTD risk in early gestation (1). It should be noted, however, that human studies investigating whether other forms of folate could be as effective as FA for NTD risk reduction are lacking (24). There remains uncertainty regarding the necessity and dosage of FA supplementation beyond the window of neural tube closure, i.e., during mid and late gestation (14). This is particularly relevant given that FA must be reduced to THF to participate in folate metabolism, then the folates are interconvertible within the folate cycle. Theoretically speaking, if the other forms of folate supplement raise folate status comparably to FA in the maternal and fetal compartments, it is intuitive that they may share the same effect as FA on disease risk reduction. While the current study did not capture the periconceptional period, it provided comparative evidence regarding the efficacy of 5-MTHF vs. FA supplementation during pregnancy for maintaining folate status in both maternal and fetal compartments in mid and late gestation.

While 5-MTHF supplementation has been shown to improve serum folate levels in women of childbearing age (25), comparative studies evaluating the effect of 5-MTHF vs. FA supplementation on folate status and maternal and fetal outcomes across pregnancy remain limited (9). Most available data instead evaluated FA alone. In a 2016 study, low-dose FA supplementation (400 μg per day) did not significantly alter folate status or UMFA concentrations in maternal and cord blood compared to placebo (26). However, this finding may have limited relevance to typical prenatal FA intake, as a 2019 review found that 99% of prescription and 91% of non-prescription prenatal MVIs contained ≥800 μg FA per serving, an amount substantially higher than the 400 μg dose that is currently recommended for women of childbearing age and the amount used in the aforementioned study (27). Several RCTs have demonstrated that 5-MTHF is at least as effective as FA in lowering plasma homocysteine levels (4, 28). A small number of studies have also examined the use of 5-MTHF vs. FA in fertility treatment and pregnancy loss, finding that 5-MTHF provided greater protection against pregnancy loss than FA (29, 30). The present study's findings build upon the literature comparing 5-MTHF to FA by providing longitudinal tracing of folate status in response to supplementation with these two forms of folate during the latter two-thirds of pregnancy, a pattern consistent with prenatal MVI use in pregnant women in the U.S. population as many women take prenatal MVIs through the duration of their pregnancy (31). The folate content in the FA-MVI was about 30% higher than that in the MTHF-MVI, yet there was no difference in 5-MTHF and total folate status across maternal blood, placenta, and fetal blood between the two groups, indicating that 6S-5-MTHF provided in a MVI supplement is at least as effective as FA-containing MVI in maintaining maternal and fetal folate status. Further, the similar effectiveness on folate status maintenance was not modified by a major folate genetic polymorphism (*MTHFR* C677T) or differential exposure to other nutrients (e.g., other B vitamins and vitamin D) in the two MVIs. Our results were consistent with findings from a similar RCT conducted on a Canadian cohort, where supplementation of FA vs. 5-MTHF for 16 weeks yielded similar folate concentrations in maternal blood (14). The current study further confirms that folate status in the placenta and fetal cord blood were also not differentially affected by folate supplement forms.

The growing use of 5-MTHF in prenatal MVIs instead of FA is partly driven by concern of UMFA detection in tissues, although the clinical implications of UMFA remain unclear (23). Earlier research has reported that FA intake exceeding 200 μg per day led to detectable amounts of UMFA in blood, while, as mentioned previously, 400 μg FA per day did not alter UMFA concentrations and more recent findings indicate that trace levels of UMFA may be present in the blood of individuals not consuming FA from fortified foods or supplements (6–8, 32). Further, UMFA has been detected in the majority of blood samples from NHANES participants (32) and 93% of cord blood samples from a 2015 Canadian cohort study (33). Consistent with prior reports, the present study demonstrated that supplementation with a prenatal MVI containing FA led to UMFA elevation in both maternal and fetal compartments (33, 34). This elevation in UMFA is generally understood to be transient during periods of supplementation, mitigating the long-term concern of overaccumulaton (35). In the present cohort, UMFA was chronically elevated in maternal blood and was primarily retained in the placenta with only two cord blood samples, both from the FA-MVI group, showing detectable amounts of UMFA. Supplementation with a 5-MTHF-containing MVI significantly reduced UMFA concentrations in maternal blood and placenta, although it did not completely eliminate UMFA detection. This is likely attributable to FA intake from foods (participants in the MTHF-VMI group had an average intake of 110 μg/day of FA) due to mandatory FA fortification of enriched cereal grain products in the U.S. Despite detection of UMFA in some participants in the MTHF-MVI group, the MTHF-MVI group experienced significant decreases in serum UMFA over the study period, while UMFA concentrations remained unchanged in the FA-MVI group. These findings suggest that formulation with 5-MTHF instead of FA in prenatal MVIs may be an effective strategy to minimize UMFA's presence in maternal and fetal circulation.

It has been hypothesized that UMFA may inhibit key enzymes in the folate cycle. For example, UMFA may inhibit DHFR, a rate-limiting enzyme responsible for converting FA to THF (36). Inhibition of MTHFR, which catalyzes the conversion of 5,10-methylene-THF to 5-MTHF, a critical point of entry into the methionine cycle of one-carbon metabolism, has also been shown *in vitro* (37, 38). Further, very high dose FA supplementation (20 mg/kg diet or 10 × recommended intake) in mice has been shown to result in a pseudo-MTHFR deficiency and ensuing liver injury (38). Also in mice, high maternal FA intake (5 × recommended intake) has been shown to induce neurocognitive damages in the rodent offspring (39).

UMFA has a higher affinity to bind folate receptor 1 (FOLR1) than 5-MTHF and variable affinity to folate transporters, potentially competing with 5-MTHF for cellular uptake (23, 40). *In vitro* studies suggest that UMFA may influence cellular growth during tumorigenesis as well as natural killer cell toxicity (41–45). The current study did not identify differences in global DNA methylation in the cord blood, consistent with the notion that UMFA may not have a substantial effect on global epigenetic regulation to be observed in studies with a small sample size (23). However, targeted methylome profiling would be necessary to determine whether specific epigenetic sites are susceptible to alteration in the presence of UMFA. Overall, further studies that investigate the association between UMFA and the health of the mother-fetus dyad are needed.

Some speculation exists regarding the notion that excess folate intake, independent of UMFA levels, may be a risk factor for diseases such as cancer, cardiometabolic disorders, and abnormalities in neurodevelopmental development including autism spectrum disorder (ASD). This speculation is grounded in data from multiple epidemiological studies, although it must be considered along with the counter evidence suggesting no correlation between high folate intake and these diseases (2, 23, 46–49). Moreover, the UL for supplemental and fortified folate was established partly because it may enable nucleotide synthesis despite a deficiency in vitamin B_12_, which serves as a co-factor in the methionine cycle that converts 5-MTHF to THF. Nevertheless, this concern of folate excess masking B_12_ deficiency may be less relevant to pregnant individuals who receive a prenatal MVI which almost certainly contains vitamin B_12_. Despite the contradictory evidence, preventing pregnant women from exceeding the UL is still a reasonable precaution. This is especially pertinent in countries with mandatory FA fortification programs, as studies have found that pregnant women routinely approach or exceed the UL for FA from food and MVIs combined (50–52). The UL was established by the National Academy of Medicine (formerly the Food and Nutrition Board of the Institute of Medicine) in 1998. It applies to synthetic forms of folate from obtained from supplements, fortified foods, or a combination of the two, similar to the more recent interpretation made by the European Food Safety Authority (EFSA) (19). Regardless, significantly fewer participants in the MTHF-MVI group reached and exceeded the UL compared to the FA-MVI group as a function of the dose being higher in the FA-MVI group. Consistent with prior findings, the present study demonstrates that nearly one-third of participants in the FA-MVI group exceeded the current UL by the end of the intervention despite taking a commonly consumed prenatal MVI containing 800 μg of FA, one of the lowest doses readily available on the market. This excessive intake could be entirely prevented by taking a prenatal MVI containing a lower dose of folate. Notably, while the FA-MVI provided in the study supplied 1,330 μg DFE and the MTHF-MVI provided 1,000 μg DFE, 330 μg DFE less, total folate concentrations in maternal serum, cord blood, and placental tissue did not differ between groups. This suggests that the higher FA dose in the FA-MVI may have exceeded the body's capacity for utilization. Nevertheless, the proportion of participants with high folate levels (>20 ng/mL) did not differ between groups, suggesting that both doses likely meet or exceed adequate intakes.

This study has several limitations. Since the intervention did not capture the periconceptional period or the first trimester of pregnancy, whether supplementing 5-MTHF vs. FA during these periods has similar effects on maternal and fetal folate status and birth outcomes remains unclear. The sample size was relatively small, and only a single dosage level of 6S-5-MTHF or FA was tested in each group. Additionally, participants had relatively low background FA intake from their diets, limiting the generalizability to populations with higher prenatal dietary FA exposure. The two MVIs have dosage differences in not only folate but also other nutrients, yet this imperfect comparison reflects the complexity of the decision that pregnant individuals make regarding MVI selection in real world conditions. It also reflects that in prenatal MVIs with complex inter-nutrient interactions, 5-MTHF and FA have similar performance in maintaining maternal and fetal folate status.

In conclusion, prenatal MVIs containing 6S-5-MTHF may offer an efficacious alternative to those containing FA in the latter two trimesters of pregnancy, as they reduce concentrations of UMFA in maternal and fetal tissues, without compromising total folate status. However, additional well-designed clinical and mechanistic studies are needed to confirm long-term maternal and fetal outcomes and to inform evidence-based guidelines across diverse populations.

## Data Availability

The original contributions presented in the study are included in the article/supplementary material, further inquiries can be directed to the corresponding author.
