# Supplementary material for: Using 6S-5-methyltetrahydrofolate instead of folic acid in prenatal multivitamin reduces unmetabolized folic acid concentrations in the mother-fetus dyad: a 24-week randomized controlled trial
Source: Front Nutr. 2026 Mar 26;13:1679067. doi: 10.3389/fnut.2026.1679067 (PMC13062212; doi:10.3389/fnut.2026.1679067)
Supplement: Supplementary file 1 [file Table_1.docx]

**Supplementary Table 1.** Nutrition facts of the two prenatal multivtamin products used in the intervention and control groups, respectively.

|  | Intervention  (2 pills per serving) | | Control  (1 pill per serving) |
| --- | --- | --- | --- |
| Vitamin A |  | 770 mcg | |
| Vitamin C |  | 85 mg | |
| Thiamin |  | 1.4 mg | |
| Riboflavin |  | 1.4 mg | |
| Niacin |  | 18 mg | |
| Vitamin B6 |  | 1.9 mg | |
| Pantothenic acid |  | 6 mg | |
| Vitamin D3 | 50 mcg (2000 IU) | 25 mcg (1000 IU) | |
| Vitamin E | 7 mg | 15 mg | |
| Folate | 1000 mcg DFE | 1330 mcg DFE | |
| Vitamin B12 | 8 mcg | 5.2 mcg | |
| Biotin | 150 mcg | 30 mcg | |
| Choline | 55 mg |  | |
| Iron | 18 mg | 27 mg | |
| Iodine | 150 mcg | 150 mcg | |
| Magnesium | 32 mg | 45 mg | |
| Boron(as calcium fructoborate6) | 0.7 mg |  | |
| Docosahexaenoic Acid (DHA) | 350 mg | 200 mg (from a separate DHA pill) | |
| Vitamin K | 90 mcg | 90 mcg | |
| Calcium |  | 250 mg | |
| Zinc |  | 11 mg | |
